# Supplementary material for: The prevalence and socio-demographic associations of household food insecurity in seven slum sites across Nigeria, Kenya, Pakistan, and Bangladesh. A cross-sectional study
Source: PLoS One. 2022 Dec 30;17(12):e0278855. doi: 10.1371/journal.pone.0278855 (PMC9803099; doi:10.1371/journal.pone.0278855)
Supplement: S1 Table — (DOCX) [file pone.0278855.s001.docx]

**S1 Table . Missing values and final sample sizes for the included variables**

| Variable | Original sample size | Missing values | Recoded to missing | Final sample size |
| --- | --- | --- | --- | --- |
| Site | 6,546 | 0 |  | 6,546 |
| Food insecurity | 6,546 | 1 |  | 6,545 |
| Highest level of school attended (household head) | 6,546 | 0 | ‘Other’ (8)  ‘Don’t know’ (41)  ‘NA/Refused to answer’ (104)  Non-defined category values (87) | 6,306 |
| Wealth quintile | 6,546 | 1 |  | 6,545 |
| Currently working | 6,546 | 56 | ‘NA’ (56) | 6,490 |
| Age of the household head | 6,546 | 0 | Outlier (1) | 6,545 |
| Sex of the household head | 6,546 | 0 |  | 6,546 |
| Household size | 6,546 | 0 |  | 6,546 |
| Number of children under 12 in the household | 6,546 | 0 |  | 6,546 |
| Percentage of adult female household members (mean) | 6,546 | 0 |  | 6,546 |
| Migration status of the household head | 6,546 | 67 | ‘NA’ (56)  ‘Don’t know’ (11) | 6,479 |
| Separate kitchen | 6,546 | 155 |  | 6,391 |
| Number of people per room used for sleeping | 6,546 | 1 |  | 6,545 |
| Possession of agricultural land (any member of the household) | 6,546 | 36 | ‘Don’t know’ (35) | 6,510 |
| Refrigerator | 6,546 | 0 |  | 6,546 |
| Is anything done to the water to make it safer to drink? | 6,546 | 2 | ‘Don’t know’ (2) | 6,544 |
| Total number of missing values |  |  |  | **435** |
| Final analytic sample size |  |  |  | **6,111** |

The total number of missing values is lower than the sum of missing values for the individual variables because of duplication.

NA: Not applicable
