# Supplementary material for: The prevalence and socio-demographic associations of household food insecurity in seven slum sites across Nigeria, Kenya, Pakistan, and Bangladesh. A cross-sectional study
Source: PLoS One. 2022 Dec 30;17(12):e0278855. doi: 10.1371/journal.pone.0278855 (PMC9803099; doi:10.1371/journal.pone.0278855)
Supplement: S2 Table — A) Other considered household characteristics, Frequency (%)/Mean (Standard deviation). B) Prevalence of food insecurity across other considered household characteristics, Frequency (%)/Mean (Standard deviation). (DOCX) [file pone.0278855.s002.docx]

## **S2 Table. A) Other considered household characteristics, Frequency (%)/Mean (Standard deviation)**

| Variable | | Nigeria | | | Kenya | | Pakistan | Bangladesh | Total |
| --- | --- | --- | --- | --- | --- | --- | --- | --- | --- |
|  |  | **NG1** | **NG2** | **NG3** | **KE1** | **KE2** | **PK1** | **BD1** |  |
| Cash transfer or social assistance | Yes | 5 (0·47) | 13 (1·80) | 11 (1·51) | 80 (8·00) | 22 (2·04) | 11 (1·18) | 83 (8·17) | 225 (3·44) |
|  | No | 1,065 (99·53) | 709 (98·20) | 719 (98·49) | 920 (92·00) | 1,054 (97·96) | 921 (98·82) | 933 (91·83) | 6,321 (96·56) |
| Mean number of people per room used for sleeping | | 2·66 (1·48) | 2·54 (1·51) | 3·19 (1·81) | 2·50 (1·57) | 2·21 (1·32) | 3·32 (1·61) | 3·17 (1·37) | 2·78 (1·57) |

## **S2 Table· B) Prevalence of food insecurity across other considered household characteristics, Frequency (%)/Mean (Standard deviation)**

| Variable | | Household food insecurity | |  |
| --- | --- | --- | --- | --- |
|  |  | **No** | **Yes** | **Total** |
| Cash transfer or social assistance | Yes | 143 (63·84) | 81 (36·16) | 224 |
|  | No | 3,731 (59·03) | 2,590 (40·97) | 6,321 |
| Mean number of people per room used for sleeping | | 2·66 (1·49) | 2·95 (1·66) | 2·78 (1·57) |
