# Supplementary material for: The prevalence and socio-demographic associations of household food insecurity in seven slum sites across Nigeria, Kenya, Pakistan, and Bangladesh. A cross-sectional study
Source: PLoS One. 2022 Dec 30;17(12):e0278855. doi: 10.1371/journal.pone.0278855 (PMC9803099; doi:10.1371/journal.pone.0278855)
Supplement: S3 Table — (DOCX) [file pone.0278855.s003.docx]

## **S3 Table. Model fit statistics for the pooled and site-specific logistic regression models**

|  | **Goodness-of-fit** | | **Predictive power** |
| --- | --- | --- | --- |
| **Model** | **Hosmer-Lemeshow** | **Pearson chi -square** | **McFadden R-squared** |
| Pooled | 0·636 | 0·293 | 0·186 |
| NG1 | 0·836 | 0·453 | 0·066 |
| NG2 | 0·620 | 0·312 | 0·074 |
| NG3 | 0·202 | 0·313 | 0·074 |
| KE1 | 0·481 | 0·422 | 0·078 |
| KE2 | 0·015 | 0·654 | 0·042 |
| PK1 | 0·417 | 0·267 | 0·027 |
| BD1 | 0·962 | 0·246 | 0·143 |
