# Supplementary material for: The prevalence and socio-demographic associations of household food insecurity in seven slum sites across Nigeria, Kenya, Pakistan, and Bangladesh. A cross-sectional study
Source: PLoS One. 2022 Dec 30;17(12):e0278855. doi: 10.1371/journal.pone.0278855 (PMC9803099; doi:10.1371/journal.pone.0278855)
Supplement: Attachment — Submitted filename: Response to reviewers 21st September 2022.docx [file pone.0278855.s004.docx]

Dear Faisal Abbas,

Thank you for the opportunity to respond to the reviewers’ comments on our article, reference PONE-D-22-14578.

We thank the reviewers for their careful reading of their paper and note that in responding to their queries the paper has improved. We hope you find the responses to be satisfactory. The point-by-point response follows in the Table below.

Yours sincerely,

Oyinlola Oyebode on behalf of all authors.

| Editorial comments | |
| --- | --- |
| 1. Please ensure that your manuscript meets PLOS ONE's style requirements, including those for file naming. The PLOS ONE style templates can be found at  <https://journals.plos.org/plosone/s/file?id=wjVg/PLOSOne_formatting_sample_main_body.pdf> and  <https://journals.plos.org/plosone/s/file?id=ba62/PLOSOne_formatting_sample_title_authors_affiliations.pdf> | Completed according to the requirements. |
| 2. If you are reporting a retrospective study of medical records or archived samples, please ensure that you have discussed whether all data were fully anonymized before you accessed them and/or whether the IRB or ethics committee waived the requirement for informed consent. If patients provided informed written consent to have data from their medical records used in research, please include this information. | N/A |
| 3. Thank you for stating the following financial disclosure:  This research was funded by the National Institute for Health Research (NIHR) Global Health Research Unit on Improving Health in Slums using UK aid from the UK Government to support global health research. The views expressed in this publication are those of the author(s) and not necessarily those of the NIHR or the UK Department of Health and Social Care.     Please state what role the funders took in the study.  If the funders had no role, please state: "The funders had no role in study design, data collection and analysis, decision to publish, or preparation of the manuscript."  If this statement is not correct you must amend it as needed.  Please include this amended Role of Funder statement in your cover letter; we will change the online submission form on your behalf. | Amended as suggested to:  “This research was funded by the National Institute for Health Research (NIHR) Global Health Research Unit on Improving Health in Slums using UK aid from the UK Government to support global health research. The funders had no role in study design, data collection and analysis, decision to publish, or preparation of the manuscript. The views expressed in this publication are those of the author(s) and not necessarily those of the NIHR or the UK Department of Health and Social Care.” |
| 4. Thank you for stating the following in the Acknowledgments Section of your manuscript:  This research was funded by…  However, funding information should not appear in the Acknowledgments section or other areas of your manuscript. We will only publish funding information present in the Funding Statement section of the online submission form.  Please remove any funding-related text from the manuscript and let us know how you would like to update your Funding Statement… Please include your amended statements within your cover letter; we will change the online submission form on your behalf. | The funding statement was reviewed in response to editorial comment no.3. The funding information in the acknowledgments has been removed. |
| 5. Thank you for stating the following in your Competing Interests section:  None  Please complete your Competing Interests on the online submission form to state any Competing Interests. If you have no competing interests, please state "The authors have declared that no competing interests exist.", as detailed online in our guide for authors at <http://journals.plos.org/plosone/s/submit-now>   This information should be included in your cover letter; we will change the online submission form on your behalf. | The authors have declared that no competing interests exist. |
| 6. We note that you have indicated that data from this study are available upon request. PLOS only allows data to be available upon request if there are legal or ethical restrictions on sharing data publicly. For more information on unacceptable data access restrictions, please see <http://journals.plos.org/plosone/s/data-availability#loc-unacceptable-data-access-restrictions>.  In your revised cover letter, please address the following prompts:  a) If there are ethical or legal restrictions on sharing a de-identified data set, please explain them in detail (e.g., data contain potentially sensitive information, data are owned by a third-party organization, etc.) and who has imposed them (e.g., an ethics committee). Please also provide contact information for a data access committee, ethics committee, or other institutional body to which data requests may be sent.  b) If there are no restrictions, please upload the minimal anonymized data set necessary to replicate your study findings as either Supporting Information files or to a stable, public repository and provide us with the relevant URLs, DOIs, or accession numbers. For a list of acceptable repositories, please see <http://journals.plos.org/plosone/s/data-availability#loc-recommended-repositories>.  We will update your Data Availability statement on your behalf to reflect the information you provide. | We have added the following to the data and study setting section:  “Secondary analysis was conducted of anonymised data collected by the NIHR Global Health Research Unit on Improving Health in Slums (<https://warwick.ac.uk/fac/sci/med/about/centres/wcfgh/slums)>.”  “Ethical approval allowed for anonymised data to be shared with researchers outside the specified Global Health Research Unit with the permission of the Unit’s management and financial committee before September 2021, and with permission of the Unit’s data manager since then.”  In response to the prompts:   a) At the time of applying for ethical approval, we sought approval to share the anonymised data outside the immediate research team with permission of the Unit’s management and financial committee, while the Unit was funded (until September 2021). Since then, data can be shared with the permission of the Unit’s data manager, Sam Watson [s.i.watson@bham.ac.uk](mailto:s.i.watson@bham.ac.uk). This was reflected in the participant information present prior to requesting informed consent. For this reason, we cannot make the data accessible without limitations. Please contact [s.i.watson@bham.ac.uk](mailto:s.i.watson@bham.ac.uk) for access to the data. b) Not applicable (there are restrictions imposed due to the way the ethical approvals that we sought and the participant information distributed prior to informed consent). |
| 7. We note that you have referenced (ie. Bewick et al. [5]) which has currently not yet been accepted for publication. Please remove this from your References and amend this to state in the body of your manuscript: (ie “Bewick et al. [Unpublished]”) as detailed online in our guide for authors <http://journals.plos.org/plosone/s/submission-guidelines#loc-reference-style> | We noted one personal communication (reference 23) which has been removed and one reference (32) which is now published. |
| Reviewer 1 | |
| The study provides insights on an important topic. However, the paper can be improved by considering the following: | Thank you for your helpful review, please see below for our responses to your specific concerns. |
| 1. The introduction needs to be improved. More relevant literature should be included. | In response to both your comments and those of reviewer 2, we have added more information to the introduction of the paper. This includes clarity on the provenance of the model depicted in Fig 1, what previous studies establish and where the evidence gaps are. |
| 2. The study claims that existing literature is limited in potential as the four dimensions ( depicted in figure 1) suggested by FAO are essential for categorization of food security. The present study also does not incorporate the entire channel mentioned in Figure 1 and many dimensions are ignored. The contribution of this study must be stated clearly and its relevance with Figure 1 be established. | We have now explained in the introduction how we developed Figure 1.  “As depicted by our conceptual framework in Fig 1, which is adapted from a framework published by the International Food Policy Research Institute (IFPRI) in 2006 [ref], the potential underlying mechanisms causing food insecurity in households are complex. According to the FAO, four dimensions have to be fulfilled to render a household food secure, ^20^ and these four dimensions were used to re-structure the IFPRI framework.”  We explained in the methods section the relevance of Figure 1 to our analysis plan:  “The selection of independent variables was based on the findings from the published literature and guided by the conceptual framework mentioned in the introduction (Fig 1).”  We added to the discussion in the limitations paragraph, to show that we are aware we were unable to examine all the dimensions represented in Figure 1:  “Fourth, although we were guided by our conceptual model (Fig 1), we were not able to explore all the potential factors of interest in the dataset, or all the dimensions of food insecurity, as this was secondary analysis of data collected to answer other primary questions. “ |
| 3. In logistic regressions, household size and number of children under the age of 12 are included as independent variables. These variables are collinear in nature and ideally only one of them should be used in the regressions. | As stated in the manuscript methods section  “The final decision was made after examining the candidate variables in terms of missing data, sufficient sample sizes, collinearity, and model fit.”  We tested for collinearity via correlation matrix and variance inflation factors which indicated no strong collinearity between any of the variables. Despite this, we did leave out one variable that we had considered “mean number of people per room used for sleeping” which was moderately correlated with household size and number of children under 12. This is stated in the Methods section. “Further, the number of people per room used for sleeping was not included due to collinearity concerns (S2 Table).” |
| 4. The regressions also include number of adult females in the household. The concern raised in point 3 is also valid here. The impact of presence of adult females on food insecurity of the household is not stated in the manuscript. | As above- we do not believe that collinearity was a problem in our regression analyses.  We have now added to the introduction the following to justify the inclusion of the presence of adult females in the household on food security:  “Single studies suggest that the gender of the household head and the number of adult women in a household affect food insecurity [refs].” |
| 5. Number of dependents in the house including underage children and elderly would be relevant for determining food insecurity. | Thank you for this suggestion. Although we are unable to repeat the analyses given current time and resources, this is well noted and in future we will consider the number of elderly residents alongside children under 12 as a key factor of interest. |
| Reviewer 2 | |
| 1. Please provide the background situation of each country in introduction in order to make reader understand the context and be able to compare and contrast. | We do not think it is necessary to provide background information on each country in the introduction. This is not routinely done in public health papers (although it is more commonly done for those reporting on research conducted in low- and middle-income countries- which does not seem fair. It is extremely rare to ever see the background situation of the US, the UK or other European countries included in paper introductions in any field). The reader can look up more context about a specific country elsewhere if they are not familiar with it. |
| 2. The problem statement is weak as well. | We have expanded the introduction, including clearer illustration of the problem to support the problem statement:  “Slum residence has clear links to many of the pathways to food (in)security illustrated in Fig 1. For example, educational, cultural and employment factors can affect human capital of slum household members, linked to poorer economic access to food. As an additional example, many slums are likely to be particularly vulnerable to extreme weather events, compared to other neighbourhoods [ref], which is linked to instability across the other key dimensions for food security (availability, economic and physical access, utilisation).” |
| 3. Provide ample literature evidence to support the problem. | The introduction has been expanded in response to both your and reviewer 1’s points. Some of the expanded text includes the following:  “Previous studies establish economic resources as an important factor for food security in slums [refs], with educational factors also associated with food security [refs] although not in all studies [ref]. Single studies suggest that the gender of the household head and the number of adult women in a household affect food insecurity [refs]. Other factors are little researched, such as the relationship between having a kitchen or certain household amenities and being food secure, which would be relevant to investigate in the context of high-dependence on ready-made foods in slums [refs]. There are contradictory findings relating to some factors, such as migration history [refs].  Particularly little research has been conducted on slum sites in Nigeria, we only identified one empirical study from Ibadan [ref]. Similarly, we only identified prevalence reports and one case study from Pakistan [refs].” |
| 4. Provide that whether survey instrument was adopted or adapted. Cite the references as well from where the questionnaire was made. | Updated as follows:  “The household questionnaire was developed to capture demographic and socioeconomic information such as household size or income as well as individual variables including age, sex, education, and migration status of all members.^22^ Existing questions, particularly from the Demographic and Health Surveys country-specific questionnaires were adopted where appropriate within the household survey, as well as questions from the World Health Organisation Study of global AGEing and adult health.  And further addition to the paragraph on the outcome of interest “This question was previously used in the 2014 Kenya Demographic and Health Survey questionnaire.” |
| 5. The survey design should mention the reliability and validity of the instrument. | We have added to the discussion section, firstly here:  “This study showed that prevalence of food insecurity differs greatly between slum sites and was often lower than previous study findings on similar areas. This may be due to the specific question used in the survey.”  And secondly in the limitations paragraph:  “Second, the outcome measure is limited to a single indicator which mainly assesses the economic access dimension of household food insecurity and is subject to the judgement of the surveyed participant and potential recall bias. It has also never been assessed for reliability and validity to our knowledge, but has been used in previous surveys.” |
| 6.The sampling design is missing. Inform about the population of the country and then the selected city. Also mention that how many people were surveyed from each city and why. What was the sampling strategy? | We have expanded the study design section to include more detail as follows:  “The methodology of the survey design is described in more detail elsewhere^21^. In summary, a household-based, spatially referenced, cross-sectional, retrospective survey was conducted in the seven selected slum sites. Sites were chosen by the research teams for pragmatic reasons after considering eligible sites that may the UN definition of a slum and were geographically discrete and named neighbourhoods within city boundaries. Using satellite imagery, a spatially referenced sampling frame was obtained for each site containing all geo-located households in the area of interest ^21^. People were considered a household if they were currently ‘living in the same housing unit or connected premises.’^22(p.2)^ An inhibitory sampling design with close pairs was used to generate a sample of 1200 well-dispersed households for each site, with the aim of recruiting 1000 households per site in total. Participants were surveyed between December 2018 and 2019 in Nigeria, Kenya and Bangladesh and between May 2019 and June 2020 in Pakistan. Up to three attempts to conduct each individual survey were made in case the participant was absent.^21^”.  Sampling design is described in great detail in the associated methods paper:  Improving Health in Slums Collaborative. A protocol for a multi-site, spatially-referenced household survey in slum settings: methods for access, sampling frame construction, sampling, and field data collection. *BMC Med Res Methodol* **19**, 109 (2019). https://doi.org/10.1186/s12874-019-0732-x  This was already referenced in the text. |
